# Erratum: Immunohistochemical investigation of neuronal injury in cerebral cortex of cobra-envenomed rats

**DOI:** 10.1590/1678-9199-2004000100005er

**Published:** 2020-11-09

In the article entitled “Immunohistochemical investigation of neuronal injury in cerebral cortex of cobra-envenomed rats”, DOI: 10.1590/S1678-91992004000100005, published in *Journal of Venomous Animals and Toxins including Tropical Diseases*, 10(1), 2004.

Page 1, where it was written:

RAHMY T.R.^1^; HASSONA I.A.^2^

^1^Zoology Department, Faculty of Science, Suez Canal University, Ismailia, Egypt; ^2^Biology Department, Faculty of Science, United Arab Emirates University, UAE.

It should be read:

RAHMY T.R.^1^; HASSOUNA I.A.^2^

^1^Zoology Department, Faculty of Science, Suez Canal University, Ismailia, Egypt; ^2^Zoology Department, Faculty of Science, Menoufia University, Egypt.

